# On allegations of invasive species denialism

**DOI:** 10.1111/cobi.13278

**Published:** 2019-03-13

**Authors:** David Munro, Jamie Steer, Wayne Linklater

**Affiliations:** ^1^ Centre for Biodiversity and Restoration Ecology Victoria University of Wellington P.O. Box 600 Wellington 6140 New Zealand; ^2^ Biodiversity Department Greater Wellington Regional Council Wellington New Zealand; ^3^ Department of Environmental Science, Policy and Management University of California–Berkeley CA U.S.A.; ^4^ Centre for African Conservation Ecology Nelson Mandela University Port Elizabeth South Africa

**Keywords:** discourse, facts, invasion biology, normative, science denialism, subjective, values, biología de la invasión, datos, discurso, negación de la ciencia, normativo, subjetivo, valores, 否定科学主义, 入侵生物学, 价值观, 事实, 论述, 规范性, 主观性

## Abstract

Science denialism retards evidenced‐based policy and practice and should be challenged. It has been a particular concern for mitigating global environmental issues, such as anthropogenic climate change. But allegations of science denialism must also be well founded and evidential or they risk eroding public trust in science and scientists. Recently, 77 published works by scholars, scientists, and science writers were identified as containing invasive species denialism (ISD; i.e., rejection of well‐supported facts about invasive species, particularly the global scientific consensus about their negative impacts). We reevaluated 75 of these works but could find no examples of refutation of scientific facts and only 5 articles with text perhaps consistent with one of the 5 characteristics of science denialism. We found, therefore, that allegations of ISD were misplaced. These accusations of science denialism may have arisen because invasion biology defines its subjects—invasive species—based on multiple subjective and normative judgments. Thus, more than other applied sciences its consensus is one of shared values as much as agreed knowledge. Criticisms of invasion biology have largely targeted those subjective and normative judgments and their global imposition, not the knowledge on which the discipline is based. Regrettably, a few invasion biologists have misinterpreted the critique of their values‐based consensus as a denial of their science when it is not. To make invasion biology a more robust and widely accepted science and to avoid unnecessary misunderstandings and conflicts, invasion biologists could be more accepting of perspectives originating from other disciplines and more open to values‐based critique from scholars and scientists outside their field. This recommendation applies to all conservation sciences, especially those addressing global challenges, because these sciences must serve and be relevant to communities with an extraordinary diversity of cultures and values.

## Abstract 2

*Article impact statement*: False, unsupported claims of invasive species denialism threaten diversity, interdisciplinarity, and progress in invasion biology.

## Abstract (es)

Sobre Argumentos de la Negación de Especies Invasoras

**Resumen**

La negación de la ciencia retrasa las prácticas y políticas basadas en evidencias, por lo que debería ser cuestionada. Esta negación ha sido una preocupación particular para la mitigación de los temas ambientales a nivel mundial, como el cambio climático antropogénico. Las argumentos en contra de la negación de la ciencia deben estar bien fundamentados y basados en evidencias o se corre el riesgo de mermar la confianza del público en la ciencia y en los científicos. Recientemente, se identificó que 77 trabajos publicados por académicos, científicos y escritores de ciencia contenían negaciones sobre las especies invasoras (ISD, en inglés) (es decir, el rechazo de datos bien respaldados sobre las especies invasoras, particularmente el consenso científico mundial sobre sus impactos negativos). Reevaluamos 75 de estos trabajos pero no pudimos encontrar ejemplos de refutación de datos científicos y sólo cinco de los artículos tuvieron texto quizás consistente con una de las cinco características de la negación de la ciencia. Por esto encontramos que las acusaciones de ISD eran inapropiadas. Estas imputaciones de negación de la ciencia podrían haber surgido a razón de que la biología de la invasión define a sus sujetos – las especies invasoras – con base en múltiples juicios subjetivos y normativos. Así, más que en otras ciencias aplicadas, el consenso de la biología de la invasión consiste tanto de valores compartidos como de conocimiento compartido. Las críticas hacia la biología de la invasión se han enfocado principalmente en esos juicios normativos y subjetivos y en su imposición global, no hacia el conocimiento sobre el que se basa la disciplina. Lamentablemente, unos cuantos biólogos de la invasión han malinterpretado las críticas a su consenso basado en valores como la negación de su ciencia, cuando no lo es. Para hacer de la biología de la invasión una ciencia más fuerte y con mayor aceptación y para evitar malentendidos innecesarios y conflictos, los biólogos de la invasión podrían estar más dispuestos a aceptar las perspectivas que se originan en otras disciplinas y más abiertos a las críticas basadas en valores dadas por los académicos y los científicos que están fuera de su campo de investigación. Esta recomendación aplica para todas las ciencias de la conservación, especialmente para aquellas que tratan las dificultades globales, ya que estas ciencias deben servir y ser relevantes para las comunidades con una diversidad extraordinaria de culturas y valores.

## 摘要

否定科学主义阻碍了基于证据的政策和实践, 应受到挑战。它特别关注的一个内容是应对全球环境问题, 如人类导致的气候变化。然而, 对否定科学主义的指控也必须有理有据, 否则会损害公众对科学及科学家的信任。最近, 77 篇由学者、科学家和科学作家发表的文章被认为包含入侵物种否定主义的思想（即拒绝接受关于入侵物种的确凿事实, 特别是其负面影响的全球科学共识）。我们对其中 75 篇文章进行了重新评估, 但没有发现其中存在反对科学事实的例子, 只有 5 篇文章可能符合否定科学主义五项特征中的一项。因此, 我们认为对入侵物种否定主义的指控存在不妥之处。出现这些对否定主义的谴责, 可能是因为入侵生物学对其研究对象——入侵物种的定义基于许多主观、规范性的判断。因而相比于其它应用科学, 入侵生物学中的共识既有共同价值观, 也有共享知识。对入侵生物学的批评主要针对那些主观和规范性的判断及其在全球范围内的实施, 而不是该学科基础的科学知识。令人遗憾的是, 一些入侵生物学家错误地将对其基于价值观的共识的批评看作是对科学研究的否定, 而事实并非如此。为了使入侵生物学这门学科更加健全、得到广泛接受, 同时避免不必要的误解和冲突, 入侵生物学家应更多地接受来自其它学科的观点, 并以开放的心态听取其它领域的学者和科学家基于价值观的评价。这一建议适用于所有的保护科学领域, 特别是那些应对全球挑战的领域, 因为这些科学必须服务并紧密联系文化和价值观高度多样化的群体。**【翻译: 胡怡思; 审校: 聂永刚】**

## Introduction

Science denialism is the rejection of the well‐supported facts and concepts that underpin a scientific consensus (Diethelm & McKee 2009). It has proven a barrier to implementing evidence‐based policy for the environment and human health, most notably for the treatment of human immunodeficiency virus (Kenyon 2008), and to mitigating anthropogenic climate change (Xifra 2016).

Although science denialism is not new, concerns for its prevalence and effects have grown in recent years (Otto 2012), likely aggravated by concerns about the ascendency of so‐called post‐truth politics (Higgins 2016) and perceptions that the public's trust in science is diminishing (Funk 2017). The deliberate obfuscation and denial of scientific facts and concepts is a serious and troubling issue. Where it occurs, it needs to be challenged. Nonetheless, it is important to ensure that claims of science denial are well founded and communicated evidentially, first, because of the potential odium directed at deniers and, second, because false accusations risk further eroding public trust in science and scientists.

Distinguishing denialism from critique is not always straightforward, however, especially when critique originates from scholars working in different disciplines, whether scientific or otherwise. Differences in the values, languages, and cultures of scholars and their disciplines can sometimes escalate debates more through misunderstanding than opposition. In those cases, different perspectives may be misinterpreted as the denial of facts (Sagoff 2018a).

## Conservation‐Science Denialism

Early in 2017 a new form of science denialism was ostensibly identified with the publication of “The Rise of Invasive Species Denialism” (ISD) by Russell and Blackburn (2017a). In this article, the authors describe ISD as the rejection of an apparent scientific consensus that invasive species cause harm.

The article was heavily critiqued in published responses from other scientists (Briggs 2017; Crowley et al. 2017; Davis & Chew 2017; Tassin et al. 2017), and it is not our intention to reprise the issues they raised. However, for the purposes of this article, it is significant that other scientists disputed whether ISD was actually occurring, pointing to the fact that Russell and Blackburn provided only anecdotal evidence for it (Crowley et al. 2017; Davis & Chew 2017). In reply to their critics, Russell and Blackburn (2017b) claimed “invasive species denial is increasing” but provided no evidence for this assertion.

Almost a year later, those empirical shortcomings appeared to be addressed when Ricciardi and Ryan (2017) published “The Exponential Growth of Invasive Species Denialism.” They listed 77 published media (e.g., articles, books, or videos) by 67 authors published from 1990 to 2016 as examples of ISD and described its apparently increasing trend. Sagoff (2018a), a philosopher whose work was included in their list, responded by again disputing the existence of ISD. Even if one were to accept all 77 cases as ISD prime facie, a rigorous reanalysis by Boltovskoy et al. (2018) illustrated that the reputed trend identified by Ricciardi and Ryan (2017) was the result of “cherry‐picked sources” and was “unsubstantiated and misleading.” They showed that, even if ISD exists, it was not becoming more common.

Ricciardi and Ryan (2017) reported using Diethelm and McKee's (2009) 5 criteria to identify their 77 examples of ISD media. However, when we asked Ricciardi for the text (quotations) from each published item that was used as evidence for ISD, he wrote that they did not keep an evidential list (17 and 21 May 2018). Instead, he said he and his coauthor “read and discussed each of those articles multiple times (we kept some notes, but not in all cases)” and “judged whether the article rejected scientific consensus.” In a fortuitous later meeting, Ryan explained that she, in fact, had extensive notes in support of the ISD categorizations. However, when we requested those notes Ricciardi declined our request (18 September 2018).

Given that the data were not reported and requests for them were denied, it is not possible for others to repeat Ricciardi and Ryan's (2017) method or evaluate how they applied Diethelm and McKee's (2009) criteria for science denialism. Because their method did not generate measures of observer reliability or variance, we also have no measure of subjectivity to help in the interpretation and comparison of their results with others.

These methodological shortcomings prompted us to reevaluate the 77 alleged examples of ISD. If ISD was occurring, we wanted to provide better empirical support to the debate. If it was not, we wanted to understand why false allegations of ISD had been leveled and how debates about and within invasion biology might be moderated and advanced constructively.

## Reevaluating Literature Examined by Ricciardi and Ryan

One of us (D.M.) read and reevaluated 75 of the 77 articles and books that were published in English and cited as examples of ISD by Ricciardi and Ryan (2017). D.M. was not familiar with or invested in the debate. He reevaluated the items by applying the same 5 criteria for science denialism (Diethelm & McKee 2009) that Ricciardi and Ryan (2017) reportedly used, following from Russell and Blackburn (2017a) (Supporting Information).

None refuted scientific facts related to invasive species. Furthermore, only 5 of the 75 articles contained arguments that might be consistent with the criteria for science denialism, and only 1 contained text that satisfied 2 criteria (Supporting Information). Instances of, for example, logical fallacies, selectivity of evidence, and straw men, occur at a low rate in the wider applied‐science literature, including the peer‐reviewed literature written by invasion biologists (Guiaşu & Tindale 2018). Thus, the small number of lapses we found is not evidence of intentional and orchestrated ISD.

The few examples we found could not be unambiguously identified as instances of science denialism. This was because their phraseology, especially in the context of the authors’ professions (e.g., philosophy), remained open to alternative, more benign interpretations. Indeed, our experience upon discussing the alleged examples of ISD among ourselves was to discover that evaluating a document for science denialism is a highly subjective, contextual, and values‐driven exercise. It is unsurprising then that D.M. drew very different conclusions in an evaluation of the same literature Ricciardi and Ryan (2017) examined. Clearly, attempts to quantify science denialism and its trends cannot be achieved through the methods applied by Ricciardi and Ryan (2017) (see also Boltovskoy et al. 2018). D.M.’s evaluations were likely not entirely free of bias or presupposition either, but we provide the evidence (Supporting Information) and justifications for decisions so that others can reevaluate our application of the criteria.

Our examination shows that Ricciardi and Ryan's claims of ISD are not based on sound evidence. Their data are either anecdotal and unquantified or based on subjective, unrepeatable survey methods. Our assessment is that the literature Ricciardi and Ryan (2017) examined very rarely contains text consistent with science denialism. In fact, we found no cases where scientific facts were denied. Few, if any, of the 67 scholars and scientists accused of ISD are demonstrably science deniers.

## Origins of Accusations of Science Denial

In recent years, it has become clear that better science and enhanced public trust in scientists are achieved when a diversity of perspectives is considered evidentially and in ways that represent and encourage the participation of the wider community (White et al. 2018). Unjustifiable accusations of science denialism, such as those we have identified, ostracise novel, minority, or outside perspectives. They stymie their consideration and evaluation, ultimately retarding the progress of science and its application (Guiaşu & Tindale 2018). Some reflection on why accusations of ISD have occurred, and a pathway to a more constructive discourse on invasion biology, is therefore justified. We make 2 observations to this end.

First, invasion biology is a global, conservation science whose subject, invasive species, is strongly defined by values and cultural judgments. The paramount importance of a values framework to invasion biologists is illustrated most clearly by widely used definitions of “invasive species.” The International Union for Conservation of Nature (IUCN), for example, defines them as “organisms introduced by man [sic] into places out of their natural range of distribution, where they become established and disperse, generating a negative impact” (IUCN 2008). This definition, also used by Russell and Blackburn (2017a), includes 3 subjective and contestable judgments. First, it implies a cultural judgment that people are extrinsic (alien) to nature. This is in contrast to the widely held belief that people and hence human‐assisted introductions are a part of nature (Gamborg et al. 2010). Second, deciding on a species’ natural range is also a subjective, sometimes cultural, decision. A natural range could be objectively defined and ecologically delineated as, for example, the places suitable for a species’ persistence, but then species described as invaders would be simply colonizing their natural range (Shah & Shaanker 2014). Most invasion biologists, instead, use “natural range” to describe a species’ recent historical distribution. Because biogeographic ranges are temporally dynamic, this definition is reliant on arbitrarily valuing where a species occurred at one historical time over another. Third, and most significantly, what constitutes a negative impact to some will not be so to others. “Impact” can be defined in cultural, economic, or biological terms, or any combination of these. Groups of people, scholars, and scientists will vary in how they regard and prioritize impacts even to the extent of regarding many as positive. Moreover, a definition based on harm renders the interpretation of invasive species impacts a tautology (Boltovskoy et al. 2018).

The scientific consensus described by invasion biologists is necessarily, therefore, a consensus over shared values more than it is about scientific facts (Boltovskoy et al. 2018; Sagoff 2018b). When the subject of a discipline is so strongly defined by values and cultural judgments, one should not be surprised when it generates robust critique from other scientists and scholars, especially from those outside the discipline. They have contested invasion biology's values‐based consensus, not its scientific consensus. And, even among invasion biologists, the values‐based consensus is not as widely held as it is often asserted. There is, for example, considerable counter‐evidence and debate among invasion biologists about the usefulness of harm and the emphasis on biogeographic origins to the science of species invasions (Boltovskoy et al. 2018). It is therefore not accurate to describe a consensus on invasive species as “overwhelming” (Russell & Blackburn 2017a) or “mainstream” (Ricciardi & Ryan 2017) and those with different opinions as “contrarians” or “more ideological than scientific” (Ricciardi & Ryan 2017).

Our second observation is that critiques of invasion biology largely focus on evaluating how the discipline's values influence the application and interpretation of its science and how those are imposed on others (Tassin et al. 2017). Again, contrary to those alleging ISD, scientific facts are not disputed. Indeed, most critiques of invasion biology are explicit in acknowledging that relocated species sometimes spread and grow in abundance to change ecosystems and biodiversity (e.g., Davis 2009; Marris 2011). They criticize simply where invasion biology and its scientists prescribe how different species are to be valued and treated.

Science can answer questions about where species live and where they come from, how many of them there are, what influences they have, and how they change ecosystems. But it cannot, and should not, make value judgments for all people and thus dictate other peoples’ relationships with those species. It is the value judgments that are intrinsic to invasion biology (our first point), which mean that some of its scientists are thought to overreach into prescribing answers to questions about value (Tassin et al. 2017) (our second point). This, not disputes over scientific knowledge, is the cause of the debate.

What has been labeled “invasive species denialism” is actually just critique of the values that underpin the common definition, interpretation, and application of invasion biology by some of its scientists. To critique a science for its underlying values and their imposition is not to deny its knowledge.

Considering that one side of this debate thinks their science is being denied, whereas the other is simply trying to contest the discipline's values, it is easy to see why this debate has seen little progress since last century (e.g., Lawton 1997). It is this fundamental mismatch in the arguments and intentions of the 2 sides of this debate that makes it unresolvable in its current form. However, there is a way to bring this debate closer to a resolution. And, it requires that invasion science and its scientists are inclusive, not disparaging, of others or other disciplines (Guiaşu & Tindale 2018), especially those practiced at the interface between knowledge and values.

## Interdisciplinarity and Inclusion to Advance Invasion Biology

The values that define and inform an applied science's subject, concepts, and practice need to be acknowledged and debated by its scientists. Medical science, for example, is routinely critiqued for its cultural judgments, and the values of its scientists and practitioners, and how those interact with the diversity of values held by patients (e.g., Kristeva et al. 2018; Te Karu et al. 2018; White et al. 2018). By reflecting on, and working with those values, medical science and scientists seek to modify and adapt the purpose, objectives, and outcomes of medical science to better serve its globally diverse public. A few invasion biologists, in contrast, have interpreted the values‐based critique of their discipline as antiscience (Ricciardi & Ryan 2017; Russell & Blackburn 2017a). They seem reluctant to genuinely engage in discourse about their values and cultural judgments, choosing instead when critiqued to restate their science arguments (Russell & Blackburn 2017b; Ricciardi & Ryan 2018).

We suggest that the different values and cultural judgments of the other communities that invasion biology interacts with and impacts are not being sufficiently discussed within the discipline (Guiaşu & Tindale 2018), even though some of the critique originates from other biologists (even invasion biologists) and philosophers of biological science (e.g., Sagoff 1999; Davis 2009; Davis et al. 2011; Thomas 2013; Boltovskoy et al. 2018). Instead, different values and perspectives inside and outside the discipline of invasion biology, and in the wider population, are currently regarded as wrong, threatening to their discipline's legitimacy, and an impediment to progress (Courchamp et al. 2017). Some invasion biologists, for example, have described critique from outside the discipline as less legitimate (e.g., “Within academia, it [ISD] is most often expressed by social scientists and philosophers, and far less by natural scientists”) (Ricciardi & Ryan 2017). Invasion biologists leveling accusations of ISD have also described critique as less legitimate because it is not a product of the peer‐reviewed literature (e.g., criticism is “typically expressed in forums where it avoids peer review” and “made outside the lens of scientific peer review”) (Ricciardi & Ryan 2017). And yet, as the accusatory peer‐reviewed articles by Ricciardi and Ryan (2017) and Russell and Blackburn (2017a) illustrate, peer‐reviewed publications are not free of unfounded partisan and prejudicial opinions.

In these ways, a few invasion biologists refuse to entertain the possibility that others, sometimes with different values and from different disciplines or cultures, would reach a different conclusion given the same scientific information. They present their science and proceed to apply their own, unique values to prescribing how it should be interpreted and applied. Ironically, this circumscribed approach retards a discipline's development, especially of an applied science such as invasion biology because, ultimately, many others must value it or it is useless. The inclusion of critique from scholars originating outside a discipline should therefore be particularly welcomed, not discouraged (Guiaşu & Tindale 2018). Because value judgments are also at the heart of invasion biology, the discipline's tenets should be able to be contested by any person, regardless of their background. Often, the advancement of scientific disciplines is driven by those outside the discipline because their views are not constrained by social, governmental, or resource (e.g., funding) networks or years studying the traditional thinking of the discipline (Kuhn 1970). Medicine, for example, routinely receives and responds constructively to critique from other disciplines, such as anthropology, sociology, and philosophy (Kristeva et al. 2018; Metz & Harris 2018; Panter‐Brick & Eggerman 2018).

Authors are often forced to publish bold new ideas outside the scrutiny of scientific peer review because their work may be considered too different from established knowledge to pass through the often conservative lens of established science. Although peer‐review is an important standard, literature that is not peer‐reviewed can thus provide useful new ways of interpreting and applying scientific knowledge. The humanities, social sciences, philosophy, and many more disciplines besides could make transformative contributions to invasion biology. Rather than ostracizing those who critique invasion biology, they should instead be included in the discipline's discourse to take advantage of the diversity of perspectives and approaches to biodiversity management that are emerging from a variety of disciplines (Crowley et al. 2017). Inclusion, and the open evaluation of differences, is preferable to allegations of science denialism that cause only conflict and disciplinary isolation.

## Conclusion

We have found that allegations of ISD (i.e., Ricciardi & Ryan 2017; Russell & Blackburn 2017a) are prejudicial, unfounded, and not arrived at by rigorous scientific analysis. Furthermore, they threaten to stymie constructive debates in and about invasion biology. And, the name‐and‐shame style of discourse (i.e., Ricciardi & Ryan 2017), alongside other kinds of assaults on peoples’ characters (e.g., Ricciardi & Ryan 2018), are not conducive to scientific progress in the public interest (Guiaşu & Tindale 2018).

Tolerance of different perspectives is a quality widely valued in open societies, including scientific communities. In science one accepts that a diversity of perspectives is an advantage, not a problem. It is also an ethical imperative because including minority views in scientific institutions is an expectation of modern science (White et al. 2018). The intolerance by some invasion biologists of different perspectives, made manifest in fallacious accusations of science denialism, is problematic.

Our analysis supports a conclusion that claims of ISD have proven to be calumnious. We are in agreement with others that they must stop (Crowley et al. 2017; Davis & Chew 2017; Boltovskoy et al. 2018; Guiaşu & Tindale 2018; Sagoff 2018a). The discipline of invasion biology needs to reflect on its values and engage with the diversity of perspectives held by the wider community that it must ultimately serve. The future of invasion biology depends on being inclusive of others—disciplines and people.

## Supporting information

A table quoting the text we found consistent with criteria for science denialism and our justification for their inclusion (Appendix S1) are available online. The authors are solely responsible for the content and functionality of these materials. Queries (other than absence of the material) should be directed to the corresponding author.
