# Supplementary material for: On allegations of invasive species denialism
Source: Conserv Biol. 2019 Mar 13;33(4):797–802. doi: 10.1111/cobi.13278 (PMC6850308; doi:10.1111/cobi.13278)
Supplement: Supplementary file 1 — A table quoting the text we found consistent with criteria for science denialism and our justification for their inclusion (Appendix S1) are available online. The authors are solely responsible for the content and functionality of these materials. Queries (other than absence of the material) should be directed to the corresponding author. [file COBI-33-797-s001.docx]

**SUPPLEMENTARY Table 1** Examples of text from the 75 items alleged to constitute invasive species denialism that could be interpreted as meeting one of the five criteria characteristics (Ch.) of science denialism (Diethelm & McKee, 2009). We provide a description of the characteristic, illustrative text from the example found, and our interpretive explanation.

| **Ch.** | **Description** | **Found examples** | **Explanation** |
| --- | --- | --- | --- |
|  |  |  |  |
| ***Construction of conspiracy theories*** | | | |
|  | Conspiracy theories suggest that scientists have reached a consensus not from individual scientists studying the evidence and reaching the same conclusion, but that they have been colluding in secret to push an agenda or belief. | | |
|  |  | Winograd, N., *Biological Xenophobia: The Environmental Movement’s War on Nature*. Huffington Post, 2013.  - ‘Indeed, “invasion biology” is a faux environmentalism, used to disguise the ugly truth about what is really motivating its adherents: an intolerance of the foreign that we have rejected in our treatment of one another, a biological xenophobia that seeks to scapegoat plants and animals for the environmental destruction caused by one species and one species alone: humans.’ | Winograd suggests that invasion biology is not concerned with describing biological invasion, but rather exists to provide a “scientific” scapegoat for humanity’s recklessness toward the natural world. |
|  |  | Theodoropoulos., D., *Invasion Biology – Critique of a pseudoscience.* 2003, Blythe: Avvar Books. p.144  - ‘Regulators understand that their funding is related to the perceived severity of the problems they address. The invasion "crisis" has been cynically promoted to the public by agencies seeking a larger portion of the tax revenue "pie". Tremendous sums are involved. In no case have public agencies sought to provide a complete and unbiased picture of man-aided dispersal, but have shamelessly engaged in a propaganda campaign designed to create fear.’ | Theodoropoulos accuses invasion biologists of creating hysteria for the own financial gain, rather than pursuing earnest scientific endeavours. |
| ***Use of fake experts and/or calumny*** | | | |
|  | Creating, or elevating the influence of, supposedly ‘expert’ scientists whose views are inconsistent with the scientific consensus. The tactic may also involve discrediting legitimate scientists and questioning of their motivations. | | |
|  |  | Cockburn, A., *Monsanto, glyphosate, and the war on invasive species*. Harpers Magazine, Sep. 2015. p. 58  - ‘The Department of the Interior claims that the annual tab is $120 billion. But this number comes from a 2005 report by David Pimentel, an ecologist and scholar at Cornell, whose dislike of aliens apparently extends to the human variety, as evidenced by his public opposition to both legal and illegal immigration.’ | Cockburn appears to be trying to use Pimentel’s apparently nationalistic political ideology to cast doubt over the motivations informing his science argument. |
|  |  | Theodoropoulos., D., *Invasion Biology – Critique of a pseudoscience.* 2003, Blythe: Avvar Books. p.xii  - ‘During the past decade "invader" fears have reached a fevered pitch, with a constant barrage from the media fanning the flames, and a huge volume of literature has been published, produced by scientists with a self-interest of promoting this ideology. Corporate and bureaucratic interests have intruded, pushing their agendas of profit and control. Finally, the use of invader fears to justify total human control of the natural world has shown that the ideology has reached a dangerous place’. | Here, Theodoropoulos attacks the motivations of invasion biologists, stating that they are interested in pushing a nativist ideology, not furthering our understanding of biological invasions. |
| ***Selectivity of evidence*** | | | |
|  | Cherry-picking data or articles to support a view which is not supported by the majority of published evidence | | |
|  |  | Pearce, F., *Trial by repetition.* | Pearce implies that invasion biology is based on non-factual stories, citing two examples of where invasion biologists have misrepresented or been careless with data. While his critique of these papers is justified, selecting two papers does not justify the implication that this is the standard across the entire discipline. |
| ***Creating impossible expectations of scientific research*** | | | |
|  | Some evidence is impossible to reasonably collect, and so approximations or proxies are used in their place. Even direct evidence comes with measures of uncertainty. Opponents will then use the inherent uncertainty of evidence as the basis for their counter-argument. | | |
|  |  | We found no expressions of this characteristic in the 75 articles reviewed. |  |
| ***Misrepresentation and logical fallacies*** | | | |
|  | In place of presenting their own evidence, denialists will instead intentionally mislead others or mis-interpret evidence by providing red herrings, false analogies, and straw men. | | |
|  |  | Chew, M., *Ecologists, environmentalists, experts, and the invasion of the ‘second greatest threat’* in *International Review of Environmental History.* 2015, Australian National University Press: Canberra. p. 17  - “As noted earlier, Edward O. Wilson is not an ichthyologist. He is by training a myrmecologist, a specialist in ants. What prompted him to construct an argument of such potential significance with reference to taxa so different from his invertebrate stock-in-trade?” | That someone is trained in one discipline does not mean that they are not able to contribute to another discipline, nor should their contributions be considered less meaningful, as is implied here by Chew. This statement attempts to remove legitimacy from Wilson’s argument by targeting his person rather than his claims. |
|  |  | Ball, G., *Border War*. New York Times, March 19 2006.  - ‘These botanical xenophobes say that a pristine natural state exists in our yards and that to disturb it is both sinful and calamitous.’  - ‘If anything suffocates us, though, it will be the environmentalists' narrowmindedness. Like all utopian visions, their dream beckons us into a perfect and rational natural world where nothing ever changes -- a world that never existed and never will.’  - ‘The anti-exotics argue that gardens should be populated exclusively by native plants, as if the exotics were trying to enter the flower bed illegally.’ | Ball repeatedly makes sweeping generalisations, often accusing environmentalists of xenophobic attitudes. Ball also misrepresents nativist opinions (i.e. by suggesting that “environmentalists” consider crop species as invasive and in need of removal) and provides no evidence to substantiate his claims. |
|  |  |  |  |
